# Stone of Large Size in the Bladder

**Published:** 1866-08

**Authors:** Benjamin Franklin

**Affiliations:** Philadelphia, Tennessee


ATLANTA

Medical and Surgical Journal.

NEW SERIES.

Vol. VIII. AUGUST, 1866. No. 6.

### ORIGINAL COMMUNICATIONS.

### ARTICLE I.

*Stone of Large Size in the Bladder.* By Benjamin Franklin, M. D., of Philadelphia, Tennessee.

James Carter, a boy thirteen years old, rather delicate, and of scrofulous diathesis from infancy, I have attended in several severe attacks of chronic diarrhœa, from which disease he has suffered during life. I was called to him the 25th of July, 1865, and found him suffering from what I supposed to be acute nephritis, which gave way to the ordinary treatment for that disease, leaving symptoms of diseased bladder, for which I treated him several weeks with but little benefit. Finally, beginning to suspect stone in the bladder, I accordingly introduced a large size knitting-needle, curved as a sound (not having a regular sound,) and could plainly detect, as I thought, the presence of the stone. The most urgent symptom presenting itself, in this case, was the intolerable irritation of the glans penis, causing him to pull at the prepuce until the whole organ became very much swollen and inflamed. I am inclined to think, taking this case for an example, that writers on surgery do not give this symptom as much attention as is due it; for in this case the irritation was so great that he was constantly, both while sleeping or awake, pulling at the penis, as though the act gave him the only relief he could get.

I was called often to see my patient, only that I might palliate his case, for an operation was entirely disregarded by his parents, who often expressed themselves that they had rather the boy would die than to suffer the pains and risks of an operation, believing he would die on the table. After attending him for several months in this situation, and using all my persuasive efforts to have the boy operated on—seeing that the patient was fast reaching a point at which an operation would do him no good, evinced by great emaciation, loss of appetite, vomiting, red tongue, quick, irritable pulse, and all the attendant symptoms of a case in its extremity—as a choice between life and death, his family finally agreed to have the operation performed; and I accordingly addressed a note to Dr. W. F. Westmoreland, of Atlanta, Ga., and requested him to visit the boy with me and operate, if in his opinion there was still remaining a chance to save the boy's life by an operation.

Before Dr. Westmoreland's arrival, I called my friend, Dr. McDonough, to see the case with me, and we both decided upon a stone of extraordinary size, the outlines of which could be clearly traced by pressing firmly above the pubis, and also by introducing the index finger into the rectum. By an examination through the rectum, the size seemed to be near that of an ordinary goose egg.

Dr. Westmoreland saw the boy the 11th of last March, and upon introducing the sound, became satisfied of the presence of a large sized stone; considering the case as an extreme one, he determined, only from a sense of duty as a surgeon, to give the boy a chance for his life by performing an operation upon him, expressing himself at the time that the chance for a recovery was only a possibility.

The patient being placed under chloroform, the lateral operation was performed, using the Lithotome, which instrument, in my opinion, very greatly simplifies, as well as expedites this operation. In less than half a minute from the time the first incision was made with the ordinary scalpel, at the request of Dr. Westmoreland, I touched the stone with my finger through the opening, and the stone would have been on the table in that space of time, had not the unusual size prevented it from being extracted by the simple use of the forceps. Enlarging the opening by expanding the blades of the forceps was repeatedly tried, without being sufficient to extract the stone, and finally the Lithotrite or Crusher was introduced, and an effort made to grasp it; but, to the astonishment of all, the Lithotrite would not grasp it by nearly an inch, after being drawn out to its greatest capacity. Here was an embarrassing point in the operation, and well calculated to unnerve the best of surgeons; but Dr. Westmoreland, with that self-possession which ever prompts a man who understands his business, conceived the idea of wearing away the stone by the use of the forceps, until the crusher would grasp it—by which he succeeded finally, after using time, patience and energy, in grasping the stone and crushing it to pieces, using the forceps on the fragments, and entirely removing from the bladder of this boy one of the largest stones, perhaps, for his age, on record.

The stone was estimated to be as large as a common size goose egg, filling the bladder to almost its utmost capacity. The boy, being put to bed, rested well by the use of a gentle opiate, and never had a bad symptom during the first twenty-five days after the operation; appetite became good; tongue cleaned off, and became moist; pulse soft, slow and regular; and, in short, his convalescence was uninterrupted and speedy, until the urine discharged naturally, which was on the twenty-fifth day, at which time he had a return of all the distressing symptoms of stone in the bladder, with all his former suffering attending the effort to urinate.

Being sent for, I concluded the difficulty consisted in inflammation of the urethra near the bulb, and accordingly addressed my treatment with but little relief. The idea occurred to me that a fragment may have been driven into the bulb of the urethra by the use of the syringe, and with this in view I recommended warm hip bath, while under active diuretics, and continued the bath until relaxations took place, and recommended the boy to make a strong effort to urinate, which he did, and succeeded in discharging a fragment of stone as large as a small pea, followed by instant relief and an immediate return of a speedy and uninterrupted convalescence.

Just here is a practical point connected with this case, that I think would be well to call the attention of the profession to, which is, the importance and necessity of always, after the crusher is used and bladder cleansed, passing a sound or catheter through the urethra, down to the seat of operation, so as to force out any fragment that might, as in this case, have become lodged in the urinary passage, and give great trouble to the physician, as well as greatly endangering the life of the patient.

Since the discharge of this fragment, the boy has had no further difficulty in any respect, and is now going to school, playing cheerfully with his schoolmates, and bids as fair to become a blessing to his parents as any member of the family.

